# Efficacy of Regular Exercise During Pregnancy on the Prevention of Postpartum Depression: The PAMELA Randomized Clinical Trial

**DOI:** 10.1001/jamanetworkopen.2018.6861

**Published:** 2019-01-04

**Authors:** Carolina de Vargas Nunes Coll, Marlos Rodrigues Domingues, Alan Stein, Bruna Gonçalves Cordeiro da Silva, Diego Garcia Bassani, Fernando Pires Hartwig, Inácio Crochemore Mohnsan da Silva, Mariângela Freitas da Silveira, Shana Ginar da Silva, Andréa Dâmaso Bertoldi

**Affiliations:** 1 Postgraduate Program in Epidemiology, Federal University of Pelotas, Pelotas, Rio Grande do Sul, Brazil; 2 Postgraduate Program in Physical Education, Federal University of Pelotas, Pelotas, Rio Grande do Sul, Brazil; 3 Department of Psychiatry, University of Oxford, Oxford, United Kingdom; 4 Centre for Global Child Health, The Hospital for Sick Children, Toronto, Ontario, Canada; 5 Medical Research Council Integrative Epidemiology Unit, University of Bristol, Bristol, United Kingdom

## Abstract

**Importance:**

Interventions to reduce postpartum depression have mainly focused on enhancing screening to increase treatment rates among women. Preventive approaches are timely from a population health perspective, particularly in low- and middle-income countries where access to mental health services is limited.

**Objective:**

To assess the efficacy of regular exercise during pregnancy on the prevention of postpartum depression.

**Design, Setting, and Participants:**

This randomized clinical trial examines a prespecified secondary outcome of the Physical Activity for Mothers Enrolled in Longitudinal Analysis (PAMELA) Study, a parallel-group, randomized clinical trial. This trial was nested in the 2015 Pelotas (Brazil) Birth Cohort Study. Between August 27, 2014, and March 14, 2016, pregnant women between 16 and 20 weeks of gestation with no contraindications to exercise were randomized 1:2 to the intervention group or control group via computer-generated randomization using a block size of 9. Data were analyzed from March 7 to May 2, 2018.

**Interventions:**

Participants assigned to the intervention were engaged in a 16-week supervised exercise program including aerobic and resistance training delivered in 60-minute sessions 3 times per week.

**Main Outcomes and Measures:**

Postpartum depressive symptoms were assessed with the Edinburgh Postnatal Depression Scale 3 months after birth. A score of 12 or greater was defined as screening positive for postpartum depression. Primary analysis was performed on a complete case basis (90% of participants who had the primary end point ascertained).

**Results:**

A total of 639 participants (mean [SD] age, 27.1 [5.1] years; mean gestational age, 16.5 [1.5] weeks) were randomly assigned to the intervention group (n = 213) or control group (n = 426). Compliance with the protocol, defined as having engaged in at least 70% of exercise sessions, was low (40.4%). There was no significant difference in mean (SD) scores for postpartum depression between the intervention group (4.8 [3.7]) and the control group (5.4 [4.1]) (mean difference, −0.6; 95% CI, −1.3 to 0.1). There was also no significant difference in rates of postpartum depression between the intervention group (12 of 192 [6.3%]) and the control group (36 of 387 [9.3%]) (odds ratio, 0.65; 95% CI, 0.33-1.28). Instrumental variable analysis indicated that noncompliance may have attenuated the effect estimates obtained in the primary analysis.

**Conclusions and Relevance:**

Moderate-intensity exercise during pregnancy did not lead to significant reductions in postpartum depression. However, noncompliance to the intervention protocol was substantial and may have led to underestimations of the possible benefits of exercise. The point estimates for this study are in the same direction as the previous randomized clinical trial on this topic. Future studies on how to promote regular exercise during pregnancy to improve compliance, particularly targeting young and less educated women, are warranted before further trials are undertaken.

**Trial Registration:**

ClinicalTrials.gov Identifier: NCT02148965

## Key Points

**Question:**

Does regular exercise during pregnancy prevent postpartum depression?

**Findings:**

In this randomized clinical trial of 639 pregnant women, individuals allocated to receive the exercise program did not have significant reductions in postpartum depression. However, noncompliance with the intervention protocol was substantial and may have resulted in underestimation of the possible benefits of the treatment (as suggested by the instrumental variable analysis).

**Meaning:**

Future studies on how to promote regular exercise during pregnancy, particularly targeting young and less educated women, are warranted before further trials are undertaken.

## Teaser

This randomized clinical trial examines the efficacy of regular exercise during pregnancy for preventing postpartum depression.

## Introduction

Interventions to reduce postpartum depression have mainly been focused on enhancing screening to increase treatment rates among women.^1^ However, their effectiveness in low- and middle-income countries with limited access to mental health services and treatment resources is currently unknown.^1,2^ Preventive strategies are timely from a population health perspective.^2,3,4^ Current evidence supports the use of exercise interventions in the management of maternal depressive symptoms during the perinatal period.^5,6,7^ Encouraging findings on the effectiveness of exercise in reducing antenatal depression have been found in a systematic review and meta-analysis of randomized clinical trials (RCTs).^7,8^ Because antenatal depression is an important predictor of postpartum depression,^9^ it has been suggested that antenatal physical activity may be a promising approach to preventing postpartum depression.^10^ However, most studies on this topic to date are observational, and further RCTs are needed to confirm the findings. The aim of this study was to assess the efficacy of a 16-week exercise intervention during pregnancy on the prevention of postpartum depression using data from a large RCT.

## Methods

### Design and Participants

We report on a prespecified secondary outcome of the Physical Activity for Mothers Enrolled in Longitudinal Analysis (PAMELA) Study, a prospective parallel-group RCT aimed at evaluating the effects of exercise during pregnancy on maternal and child health outcomes. The study was approved by the Physical Education Ethics Committee from the Federal University of Pelotas. Written informed consent was obtained from participants prior to participation. Detailed information on the trial design and recruitment can be found in Supplement 1.^11^ Findings regarding the primary outcomes of the trial (preterm birth and preeclampsia) have been published elsewhere.^12^ The current study followed the Consolidated Standards of Reporting Trials (CONSORT) reporting guideline.

The trial was nested in the 2015 Pelotas (Brazil) Birth Cohort Study, a population-based cohort of all live births from mothers living in the urban area of the city of Pelotas, Rio Grande do Sul State, Brazil, in 2015.^13^ Participants were recruited during the antenatal period of the cohort study. All women attending public and private antenatal care health services with confirmed pregnancy and estimated delivery dates between January 1 and December 31, 2015, were eligible for the cohort study.

Pregnant women eligible for the RCT were those who agreed to participate in the cohort study and who were between 16 and 20 weeks of gestation. Eligibility criteria were assessed based on information from the first antenatal interview of the cohort that was conducted before 16 weeks of gestation.^13^ Exclusion criteria included age less than 18 years, hypertension, diabetes or heart disease diagnosed prepregnancy, previous preterm birth or miscarriage, current pregnancy conceived by in vitro fertilization, multiple pregnancy, persistent bleeding, severe obesity (body mass index [calculated as weight in kilograms divided by height in meters squared] >35), heavy smoking (>20 cigarettes/day), and being currently active during leisure time (physical activity ≥150 minutes/week). The background characteristics evaluated in the present study included maternal age, gestational age, self-reported skin color (commonly used in Brazil as a proxy of ethnic/racial background for historical reasons), marital status, planned pregnancy (yes/no), schooling (maternal years of formal education), family income (minimum monthly wages), parity (total number of live births, including the current), depression before pregnancy (self-reported), prepregnancy body mass index (based on self-reported height and weight), prepregnancy leisure-time physical activity (sum of minutes in a typical week), and smoking during the first trimester of pregnancy.

To achieve the required sample size, we extended recruitment of trial participants beyond 2015 to those with expected delivery dates up to March 2016. The additional sample of 41 pregnant women recruited to the study fulfilled the same eligibility criteria. Follow-up visits to assess the outcomes of the trial were conducted in the same way as the cohort follow-up visits.

### Randomization and Blinding

Eligible participants were invited by telephone to enroll in the RCT. Recruitment of participants occurred from August 27, 2014, through March 14, 2016. A total of 639 pregnant women were randomly allocated in blocks of 9 into either intervention or control group by using a computerized random number generator, ensuring a 1:2 allocation ratio. Each block, therefore, resulted in the allocation of 3 women to the intervention group and 6 women to the control group. Sequence generation and assignment of participants to the trial groups were made by a study staff member not involved in the intervention delivery, data collection, or data analysis. Participants were enrolled in the study by staff not involved in the randomization process.

The intervention group followed a structured supervised moderate-intensity exercise protocol for 60 minutes 3 times per week. The exercise protocol was planned according to the American College of Obstetricians and Gynecologists recommendations.^14^ Participants in the control group were advised to maintain their usual daily activities, completed the same assessments as the intervention group, and were followed up according to the cohort study protocol (pregnancy, birth, and 3-month follow-ups). Because of the behavioral nature of the intervention, blinding of participants and study staff delivering the intervention (exercise instructors) was not possible. Researchers assessing the outcomes and analyzing the data were masked to group assignment.

### Procedures

The exercise facilities of the Physical Education School at the Federal University of Pelotas were used for intervention delivery. The intervention started between the 16th and 20th week of gestation and lasted at least 16 weeks (32-36 weeks of gestation). Sessions were guided and supervised by a team of 5 exercise instructors with at least 1 year of postgraduate experience. A protocol-specific training was provided to them before the start of the intervention. A set of exercises was planned and suggested for each workout, including aerobic activities, strength training, and pregnancy-specific floor exercises. The intensity of exercises was set at moderate according to the women’s perceived effort within the range of 12 to 14 on the Borg scale of perceived exertion, in which 1 is considered light effort and 20 is considered heavy effort.^15^

Exercise routines were scheduled according to the convenience of each participant and delivered in 3 training stages ensuring progressive overload until the end of the intervention. Stage 1 (weeks 1-4) included 15 minutes of aerobic activity and 35 minutes of strength and floor exercises (3 sets of 12 repetitions); stage 2 (weeks 5-10), 20 minutes of aerobics and 30 minutes of strength and floor exercises (3 sets of 10 repetitions); and stage 3 (week 11 onward), 25 minutes of aerobics and 25 minutes of strength and floor exercises (3 sets of 8 repetitions). All sessions were preceded by a 5-minute warm-up exercise and ended with a 5-minute cooldown exercise that included passive and active stretching exercises. Detailed descriptions of each exercise can be found in the trial protocol.^11^ Participants trained in groups of up to 3 per exercise instructor.

To improve compliance with the intervention, door-to-door transportation and appropriate clothes and shoes for exercising were offered to participants in the intervention group. Compliance with the intervention protocol was defined as a minimum attendance of 34 of 48 sessions (70%) during at least 16 weeks of the intervention and assessed by the percentage of sessions completed, from the start of the trial until the participants decided to stop (before or after 16 weeks). Noncompliant participants were encouraged to resume exercising until they formally declared the desire to stop the intervention.

### Outcomes

Self-reported postpartum depressive symptoms were assessed with the Edinburgh Postnatal Depression Scale (EPDS) 3 months after delivery. The EPDS is a postpartum depression screening instrument that has been extensively used in research.^16^ The scale consists of 10 items scored on a 4-point Likert scale (0-3) addressing common depressive symptoms experienced in the preceding week. A composite score is calculated by taking the sum of all items, ranging from 0 (absence of depressive symptoms) to 30 (highest score). In the present study, depressive symptoms were analyzed both as a continuous variable, by comparing mean EPDS scores between the intervention and control group, and as a binary variable, by comparing the women scoring above or below a cutoff point on the EPDS.^17^ A cutoff of 12 or more points in the scale was defined as screening positive for postpartum depression. This cutoff point has been shown to have a sensitivity of 65.4% (95% CI, 55.4%-74.4%) and specificity of 82.1% (95% CI, 77.1%-86.5%) for depression diagnosed by clinical interviews taken as the criterion standard in a previous study in this community (2004 Birth Cohort Study).^18^ The EPDS was administered as an interview by a trained interviewer.

An exploratory analysis was performed to evaluate the impact of the intervention on antenatal depressive symptoms. Symptoms were assessed in the full cohort at midpregnancy using the EPDS.^13^ The mean (SD) gestational age was 21.5 (1.6) weeks, approximately 5 weeks after the start of the intervention. It was decided to include the antenatal depression assessment as an exploratory outcome after the trial commenced given its relevance on the pathway linking exercise to the prevention of postpartum depression.

### Statistical Analysis

The sample size of the trial was calculated a priori based on an estimated risk reduction of at least 30% on the primary outcomes (preterm birth and preeclampsia).^11^ Assuming a 14% prevalence of postpartum depression (EPDS score ≥12) in the overall cohort population (data not shown), this study would have 80% statistical power to detect a minimum reduction of 47.9% (6.7 percentage points) in the prevalence of postpartum depression in the intervention group as compared with the control group with an α level of .05 using a 2-sample test.

Data analysis was primarily performed on a complete case basis (population with primary end point ascertained). Baseline characteristics were compared between the study groups and described using means and SDs for continuous variables and percentages for categorical variables. Differences in mean EPDS scores between groups were estimated using linear regression. A logistic regression model was used to estimate the effect of the intervention (odds ratios [ORs] and 95% CIs) on the rates of postpartum depression (EPDS score ≥12) using women in the control group as the reference. The same statistical procedures were used for the exploratory analysis with antenatal depressive symptoms as the outcome variable.

As a sensitivity analysis, we used multiple imputation methods to assess the impact of missing outcome data. Missing values in the postpartum EPDS scores were replaced by imputed values using chained equations with the predicted mean matching method based on the baseline explanatory variables and randomization groups.^19^ Outcome for a study participant without primary end point data was therefore imputed based on outcomes of study participants with similar baseline characteristics in the same intervention group, providing a full intention-to-treat analysis.

Data on the exercise sessions attended were used to quantify noncompliance in the intervention group. For this, those who attended at least 70% of the exercise sessions were classified as compliers. Because we observed high levels of noncompliance, we conducted a further analysis to assess whether intention-to-treat treatment effect estimates were affected by noncompliance. We therefore repeated the analysis described in an instrumental variable framework^20^ using treatment assignment as the instrument and attendance at least 70% of the sessions as the exposure (see eAppendix in Supplement 2 for a detailed description of the core assumptions and a discussion about their plausibility in our study). Our study design implies 1-sided noncompliance because participants in the control group did not have access to the exercise sessions offered to the intervention group. For this reason, our instrumental variable estimates can be interpreted as the complier average treatment effect, ie, the average causal effect of attending at least 70% of the sessions among those who were compliant.

Statistical significance was set at 2-sided *P* value less than .05. An outline of the analysis strategy is provided in the trial protocol.^11^ All statistical analyses were conducted using Stata statistical software version 13.1 (StataCorp) and R statistical software version 3.5.1 (R Core Team). Data were analyzed from March 7 to May 2, 2018.

## Results

Between August 27, 2014, and March 14, 2016, 2902 pregnant women from the cohort study were assessed for eligibility (Figure). Among the 1561 eligible women identified, 598 agreed to take part in the RCT. Another 41 participants were enrolled in the study, totaling 639 randomized participants (213 randomly assigned to the intervention group and 426 to the control group). In the intervention group, 127 participants did not meet adherence criteria. Among the study participants, a total of 60 (9.4%) were lost to follow-up or did not complete the postpartum EPDS, 21 (9.9%) in the intervention group and 39 (9.2%) in the control group. Therefore, 192 participants in the intervention group (82 adherent and 110 nonadherent) and 387 in the control group were included in the overall analysis.

**Figure.**
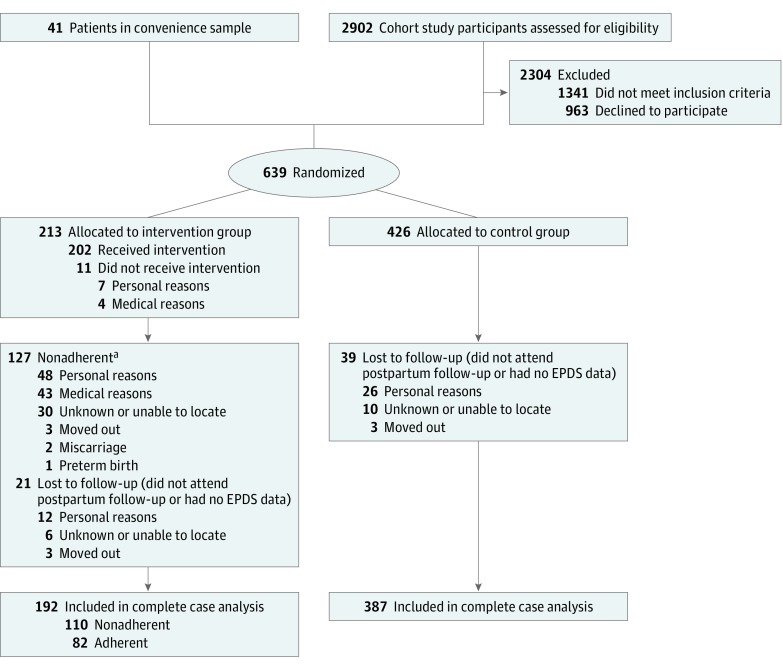
Flow Diagram of the Physical Activity for Mothers Enrolled in Longitudinal Analysis Study Participants were considered *unknown or unable to locate* as a reason for noncompliance if they stopped answering the telephone calls to schedule the weekly exercise sessions and if their reasons for nonadherence were therefore unknown. Participants were considered *unknown or unable to locate* as a reason for missing data if they did not answer phone calls to schedule the 3-month follow-up interview. EPDS indicates Edinburgh Postnatal Depression Scale. ^a^ Seventeen participants were also lost to follow-up.

Baseline characteristics of trial participants were similar between the study groups (eTable 1 in Supplement 2). The same was observed for those with complete follow-up data (Table 1). Regarding compliance to the exercise protocol, a mean (SD) of 27.1 (17.2) exercise sessions were completed (56.3%), and 40.4% (86 of 213) of participants met adherence criteria. Noncompliance in the intervention group was higher among young and less educated women (Table 2). There were no serious or unexpected adverse events reported during or after training sessions.

**Table 1.** Baseline Characteristics of Study Participants Included in the Complete Case Analysis^a^

Maternal Characteristic	IG (n = 192)	IG Missing (n = 21)	CG (n = 387)	CG Missing (n = 39)	*P* Value^b^
Age, mean (SD), y	27.2 (5.5)	26.0 (4.9)	27.3 (5.5)	25.8 (5.8)	.79^c^
Gestational age, mean (SD), wk	16.5 (1.6)	16.2 (1.3)	16.4 (1.5)	16.1 (1.3)	.78^c^
Skin color, No. (%)					
White	153 (79.7)	7 (53.9)	300 (77.5)	28 (73.7)	.59^d^
Black and mixed	39 (20.3)	6 (46.1)	87 (22.5)	10 (26.3)	
Married or living with a partner, No. (%)	173 (90.1)	17 (85.0)	352 (91.0)	32 (82.1)	.76^d^
Planned pregnancy, No. (%)	110 (57.3)	7 (35.0)	241 (62.3)	23 (59.0)	.28^d^
Multiparous, No. (%)	67 (34.9)	4 (30.8)	128 (33.1)	6 (30.0)	.66^d^
Family income, No. (%)^e^					
≤1	7 (3.7)	1 (7.7)	18 (4.9)	1 (5.3)	.74^d^
1.1-3.0	77 (41.2)	8 (61.5)	145 (39.1)	9 (47.4)	
3.1-6.0	65 (34.8)	2 (15.4)	136 (36.7)	7 (36.8)	
6.1-10.0	20 (10.7)	1 (7.7)	46 (12.4)	1 (5.2)	
>10.0	18 (9.6)	1 (7.7)	26 (7.0)	1 (5.2)	
Formal education, mean (SD), y	12.5 (3.6)	10.4 (3.9)	12.0 (3.3)	10.7 (2.7)	.09^c^
Previous depression, No. (%)	21 (11.8)	3 (15.0)	56 (15.6)	10 (25.6)	.24^d^
Smoking in the first trimester of pregnancy, No. (%)	20 (11.8)	5 (25.0)	32 (9.6)	4 (11.4)	.45^d^
Prepregnancy physical activity ≥150 min/wk, No. (%)	43 (22.4)	2 (15.4)	75 (19.4)	4 (20.0)	.40^d^
Prepregnancy body mass index, No. (%)^f^					
<18.5	4 (2.1)	0	8 (2.1)	2 (5.1)	.91^d^
18.5-24.9	91 (47.4)	7 (35.0)	185 (47.9)	16 (41.0)	
25.0-29.9	66 (34.4)	10 (50.0)	123 (31.2)	16 (41.0)	
≥30.0	31 (16.2)	3 (15.0)	70 (18.1)	5 (12.8)	

Abbreviations: CG, control group; IG, intervention group.

^a^ Complete case analysis: restricted to the 90% of participants with no missing data for the outcome (Edinburgh Postnatal Depression Scale).

^b^ *P* values refer to the comparisons between participants included in the complete case analysis.

^c^ *t* Test.

^d^ χ^2^ Test.

^e^ Minimum monthly wages compared to R$788.00 per month (US$220.00).

^f^ Calculated as weight in kilograms divided by height in meters squared.

**Table 2.** Baseline Characteristics of Adherent and Nonadherent Participants From the Intervention Group

Maternal Characteristic	Adherent (n = 82)	Nonadherent (n = 110)	*P* Value
Age, mean (SD), y	28.8 (5.3)	26.2 (5.0)	<.001^a^
Gestational age, mean (SD), wk	16.5 (1.7)	16.4 (1.5)	.78^a^
Skin color, No. (%)			
White	64 (78.1)	89 (80.9)	.72^b^
Black and mixed	18 (22.0)	21 (19.1)	
Married or living with a partner, No. (%)	70 (85.4)	103 (93.6)	.09^b^
Planned pregnancy, No. (%)	50 (61.0)	60 (54.6)	.38^b^
Multiparous, No. (%)	29 (35.4)	38 (34.6)	>.99^b^
Family income, No. (%)^c^			
≤1	3 (3.7)	4 (3.8)	.32^b^
1.1-3.0	27 (33.3)	50 (47.2)	
3.1-6.0	34 (42.0)	31 (29.3)	
6.1-10.0	8 (9.9)	12 (11.3)	
>10.0	9 (11.1)	9 (8.5)	
Formal education, mean (SD), y	13.5 (3.1)	11.8 (3.7)	<.001^a^
Previous depression, No. (%)	8 (10.3)	13 (13.0)	.65^b^
Smoking in the first trimester of pregnancy, No. (%)	9 (11.8)	11 (11.8)	>.99^b^
Prepregnancy physical activity ≥150 min/wk, No. (%)	16 (19.5)	27 (24.6)	.49^b^
Prepregnancy body mass index, No. (%)^d^			
<18.5	1 (1.2)	3 (2.7)	.78^b^
18.5-24.9	41 (50.0)	50 (45.5)	
25.0-29.9	26 (31.7)	40 (36.4)	
≥30.0	14 (17.1)	17 (15.5)	

^a^ *t* Test.

^b^ χ^2^ Test.

^c^ Minimum monthly wages compared to R$788.00 per month (US$220.00).

^d^ Calculated as weight in kilograms divided by height in meters squared.

Table 3 summarizes mean postpartum EPDS scores by study group, as well as the proportion of women who screened positive for postpartum depression with the relative treatment effects. Mean (SD) postpartum depression scores were 4.8 (3.7) in the intervention group and 5.4 (4.1) in the control group (mean difference, −0.6; 95% CI, −1.3 to 0.1; *P* = .11). Three months after birth, 48 participants (8.3%) screened positive for postpartum depression. There were no significant differences between study groups in the rates of postpartum depression (12 of 192 [6.3%] in the intervention group and 36 of 387 [9.3%] in the control group; OR, 0.65; 95% CI, 0.33-1.28). Sensitivity analysis using multiple imputation to deal with missing data^19^ yielded virtually identical results (eTable 2 in Supplement 2). The point estimates of complier average treatment effects estimated by instrumental variable methods were greater in magnitude than the respective point estimates obtained in our primary analysis (mean difference in EPDS scores, −1.3; 95% CI, −2.9 to 0.3; OR, 0.35; 95% CI, 0.10-1.78) (Table 3).

**Table 3.** Mean Postpartum EPDS Score and Proportion of Women With a Positive Screening for Postpartum Depression (EPDS Score ≥12) by Study Group^a^

Outcome	Complete Case				Instrumental Variable Analysis	
	Intervention (n = 192)	Control (n = 387)	Treatment Effect (95% CI)	*P* Value	Treatment Effect (95% CI)^b^	*P* Value
EPDS score, mean (SD)	4.8 (3.7)	5.4 (4.1)	−0.6 (−1.3 to 0.1)	.11	−1.3 (−2.9 to 0.3)	.11
EPDS score ≥12, No. (%)	12 (6.3)	36 (9.3)	0.65 (0.33 to 1.28)	.21	0.35 (0.10 to 1.78)	.21

Abbreviation: EPDS, Edinburgh Postnatal Depression Scale.

^a^ Treatment effects for the EPDS as a continuous variable are presented as mean group differences and as a binary variable (EPDS score ≥12) as odds ratios. In the instrumental variable analysis, treatment assignment was used as the instrument.

^b^ Average treatment effect among compliers; the treatment effect on mean EPDS scores was estimated using conventional 2-stage least-squares regression. For postpartum depression, we modified the conventional estimator slightly, using logistic regression as the second-stage regression to allow estimating odds ratios. The standard error was estimated by bootstrap (20 000 iterations) using percentile method.

Results from the exploratory analysis evaluating the effects of the intervention on antenatal depression are presented in Table 4. Mean antenatal EPDS scores were significantly lower among the intervention group compared with the control group (mean difference, −0.9; 95% CI, −1.6 to −0.1; *P* = .02). No significant differences in antenatal depression rates between study groups were observed (16 of 187 [8.6%] in the intervention group and 53 of 382 [13.9%] in the control group; OR, 0.58; 95% CI, 0.32-1.04).

**Table 4.** Mean Antenatal EPDS Score and Proportion of Women With a Positive Screening for Antenatal Depression by Study Group^a^

Outcome	Complete Case			
	Intervention (n = 187)	Control (n = 382)	Treatment Effect (95% CI)	*P* Value
EPDS score, mean (SD)	5.6 (3.8)	6.5 (4.4)	−0.9 (−1.6 to −0.1)	.02
EPDS score ≥12, No. (%)	16 (8.6)	53 (13.9)	0.58 (0.32 to 1.04)	.07

Abbreviation: EPDS, Edinburgh Postnatal Depression Scale.

^a^ Antenatal depressive symptoms were assessed as part of the antenatal follow-up of the cohort study (5 weeks after the start of the intervention on average). At this point, compliance to the exercise protocol was 72.3%. No EPDS data were available for 26 participants in the intervention group and 44 participants in the control group because they did not attend the antenatal cohort follow-up. Treatment effects for the EPDS as a continuous variable are presented as mean group differences and as a binary variable (EPDS score ≥12) as odds ratios.

## Discussion

We tested the efficacy of an entirely face-to-face supervised exercise intervention during pregnancy on the prevention of postpartum depression. No significant differences between groups were observed in the primary analysis. However, the low compliance to the exercise protocol, particularly among young and less educated women, may have led to an underestimation of the effect of exercise by the complete case approach. Indeed, instrumental variable estimates (which can be interpreted as the effect of actually attending ≥70% of the exercise sessions, as opposed to being assigned to the intervention or control group) were larger in magnitude than the respective estimates obtained in our primary analysis. Moreover, exploratory analysis on antenatal depressive symptoms measured approximately 5 weeks after the initiation of the intervention when compliance was about 70% indicated the possibility of an initial significant protective effect, consistent with the literature on this topic.^7^

There is considerable evidence that physical activity can reduce the risk of developing depression in adult populations.^4,21,22^ In a 2013 systematic review,^4^ baseline physical activity was negatively associated with a risk of subsequent depression in 25 of the 30 prospective studies included by the authors. Recently, a meta-analysis of physical activity and the incidence of depression, including 49 studies, reported an inverse association between physical activity and depression across different geographical regions and age groups.^22^ Among perinatal populations, however, there is limited research examining the relationship between physical activity and depression using a preventive approach.^10^

We know of only 1 study^23^ to date that has investigated the effects of exercise during pregnancy on the risk of developing postpartum depression using an experimental design similar to our study. Findings from this RCT carried out in Norway were similar to ours, in the sense that treatment effect point estimates were in the direction of a protective effect of being assigned to an exercise program (as opposed to receiving standard antenatal care only) but not achieving statistical significance. However, when restricting the analysis to a subgroup of women who did not exercise regularly prior to pregnancy, results were statistically significant. Limitations of this trial included a relatively short intervention period (12 weeks) and an exercise protocol consisting of only 1 weekly face-to-face supervised group session while home-based sessions were encouraged twice a week.^23^ Combining the estimates for postpartum depression (defined as an EPDS score ≥12 in our study and an EPDS score ≥13 in the previous trial)^23^ using random-effects meta-analysis yields the pooled OR of 0.59 (95% CI, 0.33-1.07; *P* = .08) and no indication of substantial between-study heterogeneity.

The potential underlying mechanisms by which exercise may protect against depression are not entirely clear from the literature. It has been suggested that the neurobiological antidepressant effects of exercise play a prominent role by inducing both acute and chronic responses.^24,25,26,27^ Some reports also suggest that leisure-time physical activity throughout pregnancy may play an important role in the prevention of postpartum depression by reducing the risk of antenatal depressive symptoms.^10^ In our study, mean antenatal EPDS scores were significantly lower among women from the intervention in comparison with the control group when compliance rates were much higher (Table 4), although this was not sustained into the postpartum period.

The strengths of this study included the large sample size, the fact that the study was carried out nested in a population-based prospective birth cohort study enabling the recruitment of a relatively diverse sample, and a high follow-up rate of participants. Because outcome data were assessed as part of the cohort study follow-up, missing data were minimized in our study (most of the nonadherent participants and those who dropped out of the RCT had their outcomes ascertained), increasing the confidence in the estimates found. Our study also improved on the previous RCT^23^ by including a longer duration of the exercise intervention and providing face-to-face supervision of exercise sessions.

### Limitations

This study has limitations. The lack of a significant effect of the intervention on the reduction of postpartum depression in the present study needs to be interpreted with some caution owing to the low compliance to the exercise protocol, which was particularly evident in the second half of the intervention period. Noncompliance is the major limitation of our study and it points to the challenges of implementing exercise programs during pregnancy even with multiple strategies to increase participation, such as transportation, offering flexible schedules, providing exercise clothing, and other incentives in place (all present in our study). Decreased physical activity levels during pregnancy, especially in the third trimester, are commonly observed even among women who exercised prior to pregnancy,^28^ and adherence has been a major challenge in many RCTs promoting exercise among pregnant women.^29^ Added to this, the trial criteria excluded pregnant women with certain characteristics associated with an increased risk of depressive symptoms (eg, heavy smokers and women presenting with pregnancy complications, such as hypertension and diabetes). As a result, the overall prevalence of depressive symptoms among the trial participants was lower in comparison with the full cohort study within which this study was nested (8.2% of the trial participants screened positive for postpartum depression compared with 14% of the cohort population). All these aspects contributed to reducing our power to detect potential significant treatment effects.

Some aspects of the exercise program should also be considered. Exercise intensity was monitored according to participants’ ratings of perceived exertion, a method that is considered effective to monitor exercise intensity during pregnancy.^14^ However, we cannot rule out that the perceived exertion was influenced by commonly held concerns regarding exercise safety during pregnancy.^30^ For this reason, the exercise intensity achieved by participants might not reflect the intensity that was intended for improvements in outcome. As far as the type of exercise is concerned, it is possible that different intervention modalities (eg, walking-based exercise, outdoor activities, aerobics of varying intensities, and self-paced exercise) could perhaps have greater antidepressant effects than a typical exercise program like the one designed in this study.

The lack of a clinical diagnosis of depression is another limitation of the study. However, the EPDS is a widely used screening tool for postpartum depression, having been validated against clinical interviews in the setting where the present study was carried out, and with good psychometric properties.^18^ It should also be noted that, although we were evaluating the effects of exercise on the prevention of postpartum depression in a healthy population of pregnant women, we did not assess depressive symptoms at baseline, and some of the participants may have been depressed from pregnancy, although there is no reason to believe this differed between groups owing to the similarity in baseline characteristics.

## Conclusions

In this study, a moderate-intensity exercise program over the second and third trimesters of pregnancy did not lead to significantly lower levels of postpartum depression. However, compliance with the intervention protocol was low, and secondary analysis indicated that our primary analysis may have underestimated the possible benefits of attending regular exercise sessions. Moreover, the intervention had a significant effect on reducing antenatal depressive symptoms (EPDS scores) in accordance with the existing literature. The point estimates from the 2 single trials on this topic were also directionally consistent. Nevertheless, statistically robust conclusions from trials large enough to attain proper statistical power despite substantial noncompliance are needed. Given the evidence of wider benefits of physical activity during pregnancy with respect to several other maternal and child outcomes (eg, lower weight gain and lower likelihood of gestational diabetes),^31^ innovative approaches to identify and address the main barriers are urgently needed, particularly for young and less educated women who were less likely to be compliant with the exercise protocol. Once these have been identified, they could be used to provide support for the development and further evaluation of community-based exercise interventions in pregnant women.

## Notes

Supplement 1. **Trial Protocol**

Supplement 2. **eAppendix.** Instrumental Variable Analysis **eReferences** **eTable 1.** Baseline Characteristics of the Intention-to-Treat Sample **eTable 2.** Mean EPDS Score and the Proportion of Women With a Positive Screening for Postpartum Depression by Study Group (Multiple Imputation)

Supplement 3. **Data Sharing Statement**
