# Supplementary material for: Efficacy of Regular Exercise During Pregnancy on the Prevention of Postpartum Depression: The PAMELA Randomized Clinical Trial
Source: JAMA Netw Open. 2019 Jan 4;2(1):e186861. doi: 10.1001/jamanetworkopen.2018.6861 (PMC6324311; doi:10.1001/jamanetworkopen.2018.6861)
Supplement: Supplement 1. — Trial Protocol [file jamanetwopen-2-e186861-s001.pdf]

TRIALS

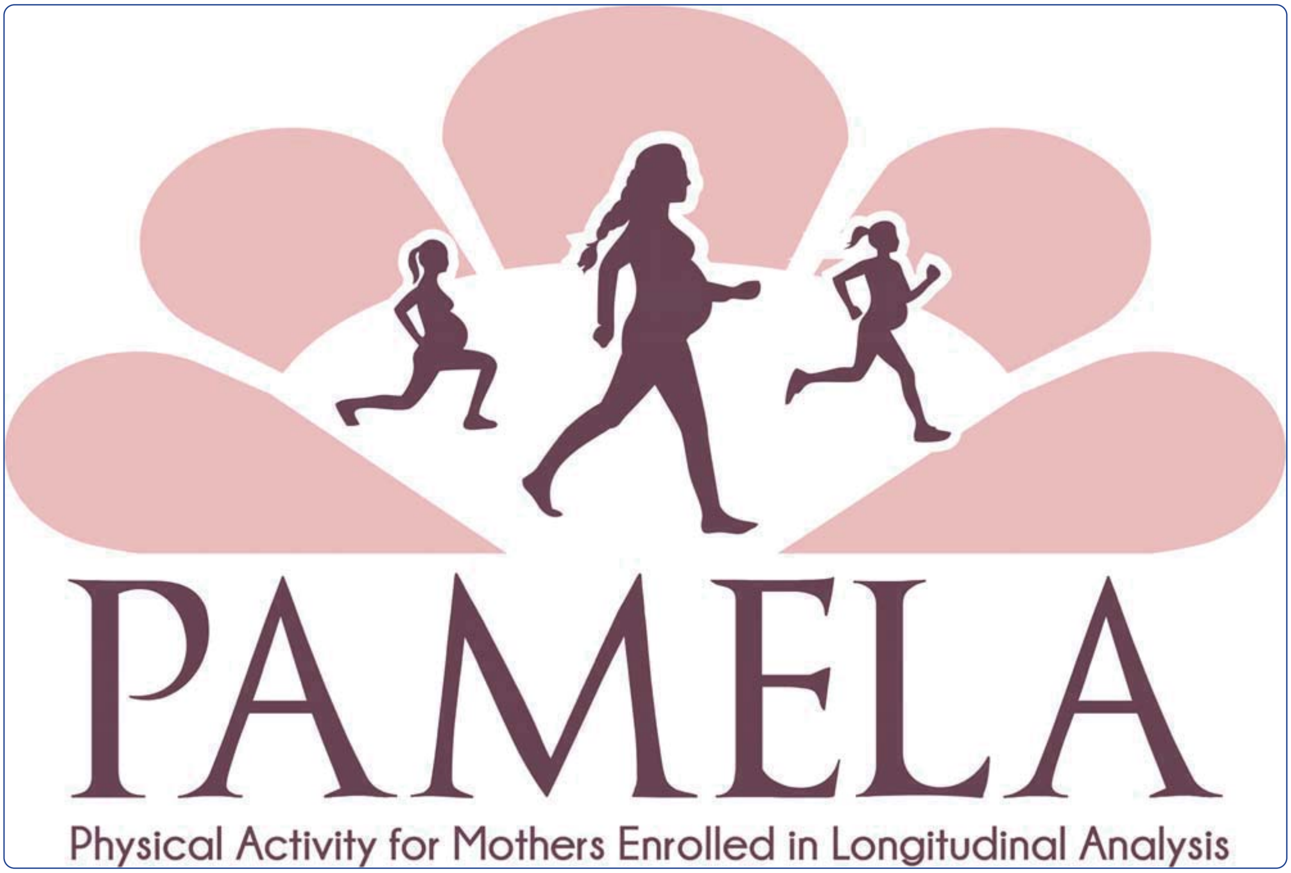


# Physical activity during pregnancy and maternal-child health (PAMELA): study protocol for a randomized controlled trial

Domingues *et al.*

BioMed Central

Domingues *et al. Trials* (2015) 16:227
DOI 10.1186/s13063-015-0749-3

Domingues et al. Trials (2015) 16:227
DOI 10.1186/s13063-015-0749-3

STUDY PROTOCOL Open Access

# Physical activity during pregnancy and maternal-child health (PAMELA): study protocol for a randomized controlled trial

Marlos Rodrigues Domingues[1,2*], Diego Garcia Bassani[3,4], Shana Ginar da Silva[5], Carolina de Vargas Nunes Coll[5], Bruna Gonçalves Cordeiro da Silva[5] and Pedro Curi Hallal[1,2,5]

## Abstract

**Background:** Preterm birth is associated with most cases of neonatal deaths and negative health outcomes, and hypertensive disorders. Hypertension is influenced by maternal behavior, such as physical activity. Physical activity is associated with better outcomes for mother and fetus, besides healthier weight gains during pregnancy. Few women are physically active during pregnancy and few clinical trials have been carried out with pregnant women. The aim of this paper is to describe the protocol of a controlled trial evaluating whether regular exercise during pregnancy may result in improved maternal-child health and neonatal outcomes.

**Methods/Design:** The PAMELA (Physical Activity for Mothers Enrolled in Longitudinal Analysis) trial is a randomized controlled trial nested in a birth cohort study. Eligible women belonging to the birth cohort will be invited (between the 16th and 20th week of gestation) to enroll in the trial. Baseline data (blood and urine samples, anthropometry and pulmonary function) will be collected at enrollment. The same assessments will be repeated eight and 16 weeks after baseline. After randomization, women will be allocated into either one of these groups: control, 426 women who will be advised to keep their usual daily activities; and intervention, 213 women who will engage in an exercise program, three sessions a week. At least 70 % attendance over 16 weeks will be required to be considered compliant to the intervention. Exercise protocol will include aerobics, strength and flexibility training. Maternal and child outcomes will be measured at the 36th week of gestation, at birth and at three, 12, 24 and 48 months postpartum. An intention-to-treat analysis will be performed.

**Discussion:** Few women are active during pregnancy and a vast majority decrease their activities or even quit exercising. We present a population-based regular exercise intervention focused on the prevention of hypertension, pre-eclampsia and preterm birth. Data on the underlying cohort will allow future analysis using different outcomes with low probability of recall bias or misclassification of exposure status. Results will potentially influence prenatal care counseling in regards to physical activity.

**Trial registration:** Clinicaltrials.gov identifier: NCT02148965, registered on 22 May 2014.

**Keywords:** Exercise, Motor activity, Randomized controlled trial, Pregnancy, Infant premature, Cohort studies, Newborn, Gestational diabetes, Pre-eclampsia, Postpartum depression

* Correspondence: marlosufpel@gmail.com
[1]Postgraduate Programme in Physical Education, Federal University of Pelotas, Rua Luís de Camões, 625, 96055630 Pelotas, Brazil
[2]GEEAF - Physical Activity Epidemiology Research Group, Rua Luís de Camões, 625, 96055630 Pelotas, Brazil
Full list of author information is available at the end of the article

© 2015 Domingues et al.; licensee BioMed Central. This is an Open Access article distributed under the terms of the Creative Commons Attribution License (http://creativecommons.org/licenses/by/4.0), which permits unrestricted use, distribution, and reproduction in any medium, provided the original work is properly credited. The Creative Commons Public Domain Dedication waiver (http://creativecommons.org/publicdomain/zero/1.0/) applies to the data made available in this article, unless otherwise stated.

## Background

The World Health Organization estimates that over 10 % of births worldwide are preterm [1]. Based on previous population studies in the city of Pelotas (Brazil), the preterm birth rate is increasing (from 6.3 to 14.7 % between 1982 and 2004) [2]. Preterm birth is associated with most cases of neonatal deaths and negative consequences throughout childhood and adult life [3, 4].

Among the strongest predictors of preterm birth are hypertensive disorders of pregnancy [5–7]. Evidence suggests that preterm birth and gestational hypertension risk may be altered by regular leisure-time physical activity [8–12]. The potential hypothesis for such a mechanism is based on reduction in blood pressure, improvement in blood lipids profile, reduced oxidative stress and inflammation reduction [13–15]. Studies also have shown that leisure-time physical activity during pregnancy is associated with lower risk of excessive weight gain [16] and better psychological health [17, 18]. Although evidence supports the benefits of physical activity during pregnancy, few women in Brazil are active during gestation, and the level of leisure-time activity decreases as pregnancy advances [19]. Further, the effect of physical activity on mother-child health outcomes is not fully understood.

With respect to different outcomes, such as diabetes, trials during pregnancy do not provide enough evidence that exercise is effective [20]. The main goal of conducting a trial in the Brazilian population is because physical activity among Brazilian women is highly associated with socioeconomic status and other characteristics not easily controlled statistically during analysis. This trial will allow for the balance of such potential confounders, as we are using a population-based sample and random allocation. Moreover, few experimental studies have been carried out to study such associations in large samples.

### Rationale

Although early studies on the effects of leisure-time physical activity during pregnancy were concerned about potential harmful effects to the health of the mother and the fetus, these have not been proven over time [21]. Current guidelines suggest that pregnant women should engage in at least 30 min of moderate-intensity physical activity on most, if not all days of the week, in the absence of medical or obstetric complications, in agreement with the recommendations for healthy adults [22].

Today, there is growing evidence supporting the association of safe physical activity during pregnancy with benefits to maternal and child health [23, 24]. However, most of the scientific evidence derives from observational studies, and there is a need for well-designed experimental studies that enable a better understanding on the impact of exercise during pregnancy on various maternal and mother-child health outcomes.

### Aim

The aim of this study is to present the experimental protocol of a trial aimed at evaluating the effectiveness of an exercise intervention, by comparing the intervention and control groups with infant outcomes assessed later in life. Data will be collected on: prematurity, gestational age, gestational weight gain, blood pressure, fasting blood glucose, postpartum weight retention, postpartum depression and birth weight.

## Methods/Design

### Study design

A randomized controlled trial will be carried out and eligible women will be sampled from the 2015 Pelotas (Brazil) Birth Cohort Study. Currently three birth cohorts (1982, 1993 and 2004) are ongoing in the city of Pelotas (southern Brazil), each with more than 4,000 subjects that were enrolled soon after birth (at hospital). The 2015 Pelotas Birth Cohort Study recruits pregnant women from health services to begin gathering data prospectively during the prenatal period. More than 3,500 women are expected to be included in the study, providing information on several health-related aspects. All women with an expected delivery date from 1 January to 31 December 2015 are eligible to be included in the cohort.

### Ethical considerations

The clinical trial protocol and the 2015 Pelotas Birth Cohort Study were submitted to the Physical Education School Ethics Committee and were approved under the numbers 649.244 and 522.064, respectively. Also, the following procedures will be followed: Participation in the study will only occur only after reading and signing the consent form; All women will be guaranteed the right to not participate in the study; We will ensure confidentiality of the collected data and document numbers (identifications, used to link databases); and Women presenting health problems during the study will be referred to appropriate health services.

The study is also registered on the Clinicaltrials.gov website under the registry number NCT02148965, entitled'Effects of exercise during pregnancy on maternal and child health: a randomized clinical trial (PAMELA)'.

### Recruitment and participants

All locations where pregnant women would potentially seek assistance during pregnancy, such as medical laboratories, ultrasound clinics, polyclinics, public primary health units, hospitals, university clinics and private obstetric and/or gynecological clinics, are being visited daily since April 2014 to identify eligible women. Based on the first prenatal interview, those women who meet inclusion criteria will be invited (by a standard phone contact) to participate in the randomized controlled trial. The recruitment

will continue during the prenatal study until we achieve the required sample size. Fig. 1 displays the study design.

### Randomization

Pregnant women meeting the inclusion criteria and who agree to participate in the randomized controlled trial will be allocated randomly into either the intervention or control group. The randomization process will be done in blocks of nine women using random software sampling. Each block will therefore result in the allocation of three women for the intervention and six women for the control group, ensuring a recruitment balance of 1:2 throughout the study. This ratio was chosen as it would minimize costs and behavioral changes in women from the remaining cohort, whilst assuring that statistical power would be unaffected. As the cohort is an observational study, our option was to expose the lowest possible number of women to the intervention. After randomization, women will be allocated into either one of these groups: control, 426 women who will keep their usual daily activities; and intervention, 213 women who will engage in a physical activity program, three sessions a week.

### Exclusion criteria

During the first contact, women will be interviewed about several aspects of their overall health, and those presenting any of the conditions described in the Table 1 will not be eligible for the trial.

### Logistics and setting

The study begins with one initial contact with the mother, prior to the 16th week of gestation, when interviewers ask for contact information, conduct a brief interview and hand a leaflet explaining the controlled trial. After enrollment in the study, women will be invited to go to the Epidemiology Research Center to collect baseline data, that is, blood and urine sampling, anthropometry, blood pressure and lung function. The same assessments (in both groups) will be repeated at eight and 16 weeks after baseline. The facilities of the Federal University of Pelotas at the Physical Education School will be used for the intervention. A second interview at hospital will take place soon after birth (when children will be measured and mothers will be asked about many aspects of their pregnancy). After these initial data collections, when children are three, 12, 24 and 48 months, interviews will be carried out at the Epidemiologic Research Center or the participant's household. Fig. 2 shows the recruitment flowchart and subsequent data collections.

### Intervention procedures: exercise protocol

The intervention will start between the 16th and 20th week of gestation and will last 16 weeks. Workouts will be guided and supervised by a team of five previously trained physical education professionals. A set of exercises will be suggested for each workout, involving aerobic activities (treadmill or stationary bike), strength training (dumbbells, machines or elastic bands) and floor exercises. The intensity of the exercises will be set according to each woman's perceived effort (within the range of 12 to 14 on the Borg Scale) [25], and will be altered according to the progress of pregnancy. The workload will change according to the week of the intervention, and the stages are described in Table 2.

Each session will last around 60 min, and will include aerobic exercises (treadmill or stationary cycling), strength training (with focus on major muscle groups and pregnancy-specific exercises to help alleviate low back pain, and work abdominal and pelvic floor muscles to prevent urinary incontinence) and will end with stretching exercises. The exercises are described in the Table 3.

### Primary outcome measures

Preterm birth (gestational age below 37 weeks of pregnancy), and pre-eclampsia (blood pressures above 140/90 mmHg and proteinuria above 15.0 mg/dL) will be assessed during pregnancy and soon after birth.

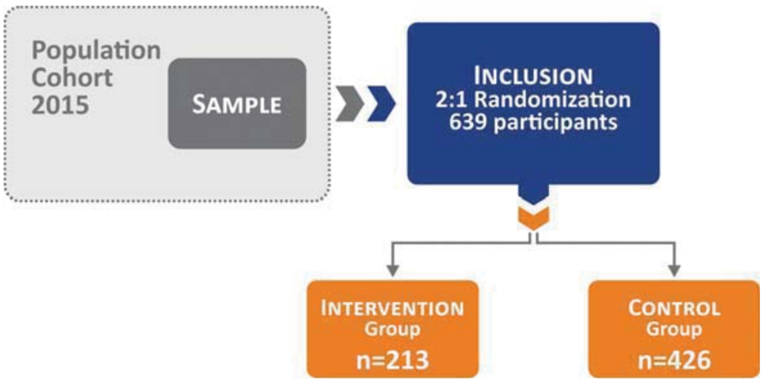


**Fig. 1** Study design

**Table 1** Exclusion criteria of the randomized controlled trial

| Exclusion criteria | Description |
|---|---|
| Age | Below 18 years |
| Hypertension | Diagnosed before pregnancy/Self-reported |
| Diabetes | Diagnosed before pregnancy/Self-reported |
| Preterm birth | History of previous preterm birth |
| Miscarriage | History of previous miscarriage |
| Heart disease | Diagnosed before pregnancy/Self-reported |
| *In vitro* fertilization | Fertilization in the current pregnancy |
| Double/twin pregnancy | Twin pregnancies confirmed by ultrasound |
| Persistent bleeding | Women presenting persistent bleeding |
| Severe obesity | Body mass index above 35 kg/m$^2$ |
| Heavy smokers | Smoking more than 20 cigarettes a day |
| Active women | Weekly leisure-time physical activity > 150 min/Self-reported |

### Secondary outcome measures

Secondary outcomes will be assessed during pregnancy and until children reach the age of 48 months, specifically: blood lipid profile, lung function (peak expiratory flow - nSpire Health PiKo-1® - nSpire Health, Inc. - 1830 Lefthand Circle - Longmont, CO 80501, USA), gestational diabetes (self-reported, by the time of delivery), gestational weight gain (calculated based on pre-gestational weight and weight at admission to deliver), mode of delivery (vaginal or cesarean section), birth weight (in grams), length at birth (in cm) and fetal growth (according to the new Intergrowth-21st fetal growth curves [26]). At three months post-partum two outcomes will be measured: post-partum weight retention (difference between current weight and pre-gestational weight) and depression (measured by the Edinburgh Postnatal Depression Scale). Infant neurodevelopment will be assessed (measured by the Battelle Developmental Inventory) at the 12, 24 and 48-month visits [27].

### Follow-up and compliance to the study

To improve the follow-up rate, at the beginning of the study participants will be informed of the importance to attend all sessions, and the staff will register name and contact information for follow-up on missed sessions to collect information on the reasons for the absence, and to offer women the opportunity to attend the session on a different day and/or time as soon as possible. Door-to-door transportation and a kit, containing a t-shirt, running tights and running shoes, will be offered for women in the intervention group. Both groups will be given study t-shirts and laboratory results around 10 days after collection. To be considered adherent to the intervention, women should attend 16 weeks of the program and a minimum of 34 (70 %) workout sessions, and cannot miss more than six training sessions in a row.

### Control group

Women allocated to the control group will be instructed to continue their usual routines, will do the same assessments as the intervention group and will be followed by the 2015 Pelotas (Brazil) Birth Cohort Study.

### Sample size calculation

For the sample size calculation, based on a statistical power of 80 %, a level of significance set at 5 % and using different outcomes from the last birth cohort [28] and estimates based on secular trends (preterm birth at 16 %, gestational hypertension at 18 %, leisure-time physical activity during pregnancy at 13 % and estimate of risk reduction with the intervention at 30 %), we estimated that 213 women would be necessary for the intervention group. The intervention:control ratio will be 1:2, therefore 426 will be included as the control group.

### Statistical analysis

Statistical analyses will be conducted on an intention-to-treat basis, but secondary analyses will be performed, including only those considered adherent to the protocol. Baseline characteristics will be presented using descriptive statistics to compare both groups.

According to the type and distribution of variables, between-groups differences will be evaluated using adequate tests. Continuous variables will be analyzed by t tests (for symmetrically distributed data), or Mann–Whitney U tests (for asymmetrical data). Categorical

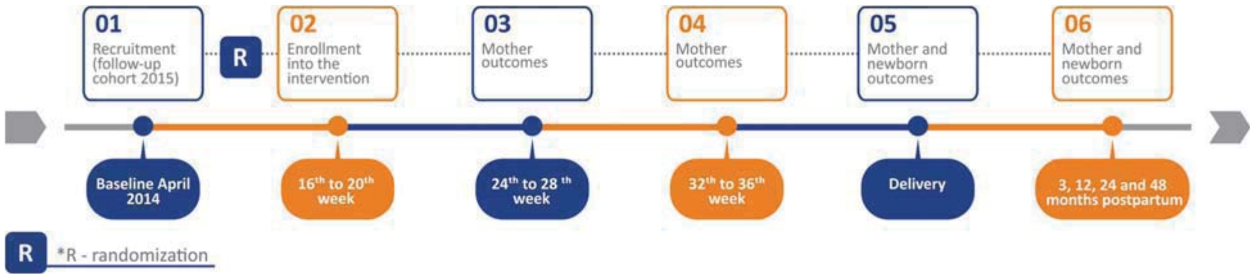


**Fig. 2** Timeline of PAMELA study

**Table 2** Stages of training routines during pregnancy according to week of intervention

| Weeks of intervention | Warm-up | Aerobic exercise | Strength training/Floor exercises | Stretching |
|---|---|---|---|---|
| 1 to 4, Stage 1 | 5 min | 15 min | 35 min (sets: 3 × 12 repetitions) | 5 min |
| 5 to 10, Stage 2 | 5 min | 20 min | 30 min (sets: 3 × 10 repetitions) | 5 min |
| 11 to end, Stage 3 | 5 min | 25 min | 25 min (sets: 3 × 8 repetitions) | 5 min |

variables will be analyzed with chi-square tests or Fisher's exact test, as appropriate. General linear model or logistic regression will be employed to control for confounding factors. More specifically, based on the perinatal study information, preterm birth, gestational diabetes and eclampsia incidences will be compared in the two groups using chi-square tests, followed by multivariable analyses when adequate; linear regressions will be carried out to evaluate potential differences in birth weight, gestational weight gain and lipid profile. Analysis will be carried out in the statistical package STATA 12.0 (StataCorp, 4905 Lakeway Drive. College Station, TX, 77845 USA) and significance will be set at 5 %.

## Discussion

Despite current evidence, few women are physically active during pregnancy in Brazil and worldwide [19, 29, 30]. As physical activity is considered a behavioral aspect of living, changes are not easily made, despite knowledge accumulation. During pregnancy, literature shows that even those women who were previously active decrease their activities, or even quit exercising [31]. This is the first large exercise intervention during pregnancy in Brazil, especially using a population-based sample. If our hypothesis is confirmed, the results of the study will be potentially used routinely to counsel pregnant women during prenatal care about physical activity during pregnancy.

Among the strengths of our study, we highlight that several efforts will be made to improve attendance to the program, such as free transportation to and from the intervention setting and material incentives (athletic apparel from the study organization). Also, our intervention program includes three weekly sessions of exercise, with a large sample size, which is not commonly found in the literature. The main limitation of any behavioral controlled trial is with respect to the exposure, especially in the control group, because we cannot guarantee that these women will not be exposed in their daily lives to some level of physical activity. However, based on previous data from this population, we know that the prevalence of regular leisure-time physical activity during pregnancy is extremely low (below 15 %) and, as we excluded from the randomization women who were previously active, we

**Table 3** Description of exercises included in the intervention during pregnancy

| Exercise type | Workout description |
|---|---|
| Aerobic exercise | Treadmill or stationary cycling (unless cycling feels uncomfortable with pregnancy evolution). |
| | Intensity of continuous exercise: at the highest comfortable intensity ('talking intensity'): the intensity will be kept at a level that allows a conversation to be carried out, that is, 12 to 14 on a 6 to 20 Borg Scale of Perceived Exertion. Women will be allowed to jog on the treadmill if desired. |
| Strength training | Weight machines: shoulder press, bench press, knee extension, seated row and hip adduction. |
| | Free weights and elastic bands exercises for all large muscle groups. |
| | Intensity of weight training: |
| | Stage 1: weights will be as heavy as the woman can lift 12 times; |
| | Stage 2: weights will be as heavy as the woman can lift 10 times; |
| | Stage 3: weights will be as heavy as the woman can lift 8 times. |
| Floor and Swiss ball exercises | Mandatory exercises: |
| | a) alternating arm/leg raise; |
| | b) ball squats; |
| | c) spinal rotation; |
| | d) Kegel contractions; |
| | e) spinal flexion (cat stretch); |
| | f) pelvic tilts on the ball. |
| Stretching | Stretching exercises will especially focus on the cervical area (neck), low back, lower leg (calves), quadriceps, pectoralis and gluteus region. Passive and active stretching will be used, according to the muscle group being stretched. |

believe that the number of active women in the control group will not bias the study or lead to any kind of misclassification.

## Trial status

Participant recruitment for this trial is ongoing. Recruitment began on September, 2014 and is expected to end by September, 2015.

### Competing interests

The authors declare that they have no competing interests.

### Authors' contributions

MRD, SGS, CVNC and BGCS worked in the planning of the trial, conducted the fieldwork and wrote the manuscript. DGB and PCH collaborated in the planning of the trial and the writing of the manuscript. All authors have read and approved the final version of the manuscript.

### Acknowledgments

This article is based on data from the 2015 Pelotas (Brazil) Birth Cohort Study. This project is funded through a New Investigator Award (grant number 095582/Z/11/Z) from the Wellcome Trust obtained by PCH (last author). The overall project is about physical activity levels, trends, correlates, health consequences, interventions and policy. The 2015 cohort project, in which the PAMELA trial is nested, was the main part of the application. The project is conducted by the Postgraduate Program in Epidemiology of the Federal University of Pelotas, Brazil with the collaboration of the Brazilian Public Health Association (ABRASCO) and is currently also supported by the Brazilian National Research Council (CNPq) and the Coordination for the Improvement of Higher Education Personnel (CAPES) (grant number 2207/2012).

### Author details

[1]Postgraduate Programme in Physical Education, Federal University of Pelotas, Rua Luís de Camões, 625, 96055630 Pelotas, Brazil. [2]GEEAF - Physical Activity Epidemiology Research Group, Rua Luís de Camões, 625, 96055630 Pelotas, Brazil. [3]Centre for Global Child Health, The Hospital for Sick Children, 555 University Avenue, Toronto, ON M5G 1X8, Canada. [4]Department of Paediatrics, Faculty of Medicine, University of Toronto, 1 King's College Circle, Toronto, ON M5S 1A8, Canada. [5]Postgraduate Programme in Epidemiology, Federal University of Pelotas, Rua Marechal Deodoro, 1160, 96020220 Pelotas, Brazil.

Received: 5 February 2015 Accepted: 7 May 2015
Published online: 24 May 2015

### References

1. World Health Organization. Born too soon: the global action report on Preterm Birth. Geneva, Switzerland: WHO; 2012.
2. Barros FC, Victora CG, Matijasevich A, Santos IS, Horta BL, Silveira MF, et al. Preterm births, low birth weight, and intrauterine growth restriction in three birth cohorts in Southern Brazil: 1982, 1993 and 2004. Cad Saude Publica. 2008;24 Suppl 3:390–8.
3. Blencowe H, Cousens S, Chou D, Oestergaard M, Say L, Moller AB, et al. Born too soon: the global epidemiology of 15 million preterm births. Reprod Health. 2013;10 Suppl 1:S2.
4. Blencowe H, Lee AC, Cousens S, Bahalim A, Narwal R, Zhong N, et al. Preterm birth-associated neurodevelopmental impairment estimates at regional and global levels for 2010. Pediatr Res. 2013;74 Suppl 1:17–34.
5. Sibai B, Dekker G, Kupferminc M. Pre-eclampsia. Lancet. 2005;365(9461):785–99.
6. Mugo M, Govindarajan G, Kurukulasuriya LR, Sowers JR, McFarlane SI. Hypertension in pregnancy. Curr Hypertens Rep. 2005;7(5):348–54.
7. Hernandez-Diaz S, Toh S, Cnattingius S. Risk of pre-eclampsia in first and subsequent pregnancies: prospective cohort study. BMJ. 2009;338:b2255.
8. Sorensen TK, Williams MA, Lee IM, Dashow EE, Thompson ML, Luthy DA. Recreational physical activity during pregnancy and risk of pre- eclampsia. Hypertension. 2003;41:1273–80.
9. Magnus P, Trogstad, Owe KM, Olsen SF, Nystad W. Recreational physical activity and the risk of preeclampsia: a prospective cohort of Norwegian women. Am J Epidemiol. 2008;168:952–7.
10. Domingues MR, Matijasevich A, Barros AJ. Physical activity and preterm birth: a literature review. Sports Med. 2009;39(11):961–75.
11. Field T. Prenatal exercise research. Infant Behav Dev. 2012;35(3):397–407.
12. Aune D, Saugstad OD, Henriksen T, Tonstad S. Physical activity and the risk of preeclampsia a systematic review and meta-analysis. Epidemiology. 2014;25:331–43.
13. Clapp JF, Kiess W. Effects of pregnancy and exercise on concentrations of the metabolic markers tumor necrosis factor alpha and leptin. Am J Obstet Gynecol. 2000;182:300–6.
14. Butler CL, Williams MA, Sorensen TK, Frederick IO, Leisenring WM. Relation between maternal recreational physical activity and plasma lipids in early pregnancy. Am J Epidemiol. 2004;160(4):350–9.
15. Oken E, Ning Y, Rifas-Shiman SL, Radesky JS, Rich-Edwards JW, Gillman NW. Associations of physical activity and inactivity before and during pregnancy with glucose tolerance. Obstet Gynecol. 2006;108(5):1200–7.
16. Streuling I, Beyerlein A, Rosenfeld E, Hofmann H, Schulz T, von Kries R. Physical activity and gestational weight gain: a meta-analysis of intervention trials. BJOG. 2011;118:278–84.
17. Robledo-Colonia AF, Sandoval-Restrepo N, Mosquera-Valderrama YF, Escobar-Hurtado C, Ramírez-Vélez R. Aerobic exercise training during pregnancy reduces depressive symptoms in nulliparous women: a randomised trial. J Physiother. 2012;58(1):9–15.
18. Teychenne M, York R. Physical activity, sedentary behavior, and postnatal depressive symptoms: a review. Am J Prev Med. 2013;45(2):217–27.
19. Domingues MR, Barros AJ. Leisure-time physical activity during pregnancy in the 2004 Pelotas Birth Cohort Study. Rev Saude Publica. 2007;41(2):173–80.
20. Yin YN, Li XL, Tao TJ, Luo BR, Liao SJ. Physical activity during pregnancy and the risk of gestational diabetes mellitus: a systematic review and meta-analysis of randomised controlled trials. Br J Sports Med. 2014;48(4):290–5.
21. Downs DS, Chasan-Taber L, Evenson KR, Leiferman J, Yeo S. Physical activity and pregnancy: past and present evidence and future recommendations. Res Q Exerc Sport. 2012;83(4):485–502.
22. Committee on Obstetric Practice. ACOG committee opinion. Exercise during pregnancy and the postpartum period. Number 267, January 2002. American College of Obstetricians and Gynecologists. Int J Gynaecol Obstet. 2002;77:79–81.
23. Prather H, Spitznagle T, Hunt D. Benefits of exercise during pregnancy. PM R. 2012;4(11):845–50. quiz 850.
24. Mudd LM, Owe KM, Mottola MF, Pivarnik JM. Health benefits of physical activity during pregnancy: an international perspective. Med Sci Sports Exerc. 2013;45(2):268–77.
25. Borg G. Perceived exertion as an indicator of somatic stress. Scand J Rehabil Med. 1970;2(2):92–8.
26. Papageorghiou AT, Ohuma EO, Altman DG, Todros T, Ismail LC, Lambert A, et al. International standards for fetal growth based on serial ultrasound measurements: the Fetal Growth Longitudinal Study of the INTERGROWTH-21st Project. Lancet. 2014;384:869–79.
27. Newborg J, Stock J, Wnek L, Guidabaldi J, Svinicki J. Battelle developmental inventory. Itasca, IL: Riverside Publishing; 1988.
28. Domingues MR, Barros AJ, Matijasevich A. Leisure time physical activity during pregnancy and preterm birth in Brazil. Int J Gynecol Obstet. 2008;103(1):9–15.
29. Evenson KR, Wen F. National trends in self-reported physical activity and sedentary behaviors among pregnant women: NHANES 1999–2006. Prev Med. 2010;50(3):123–8.
30. Amezcua-Prieto C, Lardelli-Claret P, Olmedo-Requena R, Mozas-Moreno J, Bueno-Cavanillas A, Jiménez-Moleón JJ. Compliance with leisure-time physical activity recommendations in pregnant women. Acta Obstet Gynecol Scand. 2011;90(3):245–52.
31. Gaston A, Cramp A. Exercise during pregnancy: a review of patterns and determinants. J Sci Med Sport. 2011;14(4):299–305.
