# Supplementary material for: Efficacy of Regular Exercise During Pregnancy on the Prevention of Postpartum Depression: The PAMELA Randomized Clinical Trial
Source: JAMA Netw Open. 2019 Jan 4;2(1):e186861. doi: 10.1001/jamanetworkopen.2018.6861 (PMC6324311; doi:10.1001/jamanetworkopen.2018.6861)
Supplement: Supplement 2. — eAppendix. Instrumental Variable Analysis eReferences eTable 1. Baseline Characteristics of the Intention-to-Treat Sample eTable 2. Mean EPDS Score and the Proportion of Women With a Positive Screening for Postpartum Depression by Study Group (Multiple Imputation) [file jamanetwopen-2-e186861-s002.pdf]

Supplementary Online Content

Coll CVN, Domingues MR, Stein A, et al. Efficacy of regular exercise during pregnancy on the prevention of postpartum depression: the PAMELA randomized clinical trial. *JAMA Netw Open*. 2018;2(1):e186861. doi:10.1001/jamanetworkopen.2018.6861

eAppendix. Instrumental Variable Analysis

eReferences

eTable 1. Baseline Characteristics of the Intention-to-Treat Sample

eTable 2. Mean EPDS Score and the Proportion of Women With a Positive Screening for Postpartum Depression by Study Group (Multiple Imputation)

This supplementary material has been provided by the authors to give readers additional information about their work.

© 2019 Coll CVN et al. *JAMA Network Open.*

eAppendix. Instrumental variable analysis

In the main text, we mentioned that we used instrumental variable (IV) methods to estimate the average effect of the exposure on postpartum depression (both as a continuous and as binary variable). The exposure was defined as follows: 1=attended to ≥70% of the exercise sessions; 0=attended to <70% of the exercise sessions (given the study design, everyone in the control group is classified as "0" – i.e., not exposed).

For the IV analysis, we used intervention assignment (IA) as an instrument. For IA to be a valid instrument, it must satisfy the three core IV assumptions [1,2]:

i) Relevance: IA must be associated with the exposure.

ii) Independence: IA must have no common causes with the outcome.

iii) Exclusion restriction: IA has no effect on the outcome that is not fully mediated by the exposure.

If any of these three assumptions are violated, IA is not a valid instrument and the IV analysis should not be performed. We now discuss the plausibility of each of the three assumptions above in our study.

Assumption i) is empirically verifiable. As described in the main text, 40% (95% CI 59-76) of the women in the intervention group attended to ≥70% of the exercise sessions.

Assumption ii) is not empirically verifiable. However, in the absence of selection bias, randomization of the intervention guarantees (in expectation) that IA is independent of any other causes of the outcome (and, indeed, of any other pre-randomization characteristics). Between-group comparisons regarding measured baseline characteristics provided empirical corroboration that randomization effectively balanced the intervention and control groups (eTable 1). Regarding selection bias, it is unlikely to be substantial in our study because the complete-case analysis excluded only 10% (including both those lost to follow-up and those followed-up but with no outcome data) of the initial sample, and those excluded were similar (again, regarding measured baseline characteristics) to those included (Table 1).

Assumption iii) is not empirically verifiable. However, unlike assumption ii), it is not ensured by randomization. It is possible that simply going to the exercise sessions provides mental health benefits independently on exercising or not. Examples of such potential "off-target" effects include receiving support or advice from other pregnant women or understanding that attending to the exercise sessions is good for her child, thus making the women feel better and more confident about her pregnancy and about her skills as a mother.

Here, it is important to understand the definition of the exposure variable: we defined the exposure variable as attending to the exercise sessions, not how much exercise each woman actually did in or out of those sessions. Therefore, effects such as those two outlined above are not real off-target effects, but part of the effect of attending to exercise sessions designed for pregnant women, which is a combination of exercise, interaction with other mothers, etc. Given the definition of the exposure variable, true off-target effects would be effects from IA to the outcome not mediated by attending to the exercise sessions. However, such potential psychosocial effects of IA would be true violations of assumption iii) if the analysis aims at estimating the effect of physical activity

© 2019 Coll CVN et al. *JAMA Network Open.*

itself (which is a component of “attending to exercise sessions”). This implies that designs such as ours require stronger assumptions to estimate the effect of exercise itself than the effect of attending to exercise sessions.

eReferences

1. Imbens, G., and J. Angrist (1994). Identification and Estimation of Local Average Treatment Effects. Econometrica, Vol. 61, No. 2, 467-476.

2. Greenland S. An introduction to instrumental variables for epidemiologists. Int J Epidemiol. Dec 2000;29(6):1102.

© 2019 Coll CVN et al. *JAMA Network Open.*

eTable 1. Baseline characteristics of the intention-to-treat sample. [a]

Maternal characteristics	Intervention (n=213)	Control (n=426)
Mean age, years (Mean [SD])	27.3 (5.4)	27.1 (5.6)
Mean gestational age, weeks (Mean [SD])	16.4 (1.6)	16·4 (1.5)
Skin color (N [%])		
White	160 (78.1)	316 (77.6)
Black and mixed	45 (22.0)	91 (22.4)
Married or living with a partner (N [%])	184 (89.8)	372 (91.4)
Planned pregnancy (N [%])	115 (56.1)	252 (61.9)
Multipara (N [%])	71 (34.6)	134 (32.9)
Family income, minimum monthly wages (N [%])		
≤ 1	8 (4.0)	19 (4.9)
1·1 – 3	85 (42.5)	154 (39.5)
3·1 – 6	67 (33.5)	143 (36.7)
6·1 –10	21 (10.5)	47 (12.1)
> 10	19 (9.5)	27 (6.9)
Schooling, years of formal education (Mean [SD])	12.3 (3.6)	11· 9 (3.3)
Previous depression (N [%])	24 (12.1)	66 (16.5)
Smoking in the first trimester of pregnancy (N [%])	25 (13.2)	36 (9.8)
Pre-pregnancy physical activity, 150 min/week (N [%])	45 (22.0)	79 (19.4)
Pre-pregnancy BMI, kg/m^2 (N [%])		
<18·5	4 (1.9)	10 (2.3)
18·5-24·9	98 (46.2)	201 (47.3)
25·0-29·9	76 (35.9)	139 (32.7)
≥30	34 (16.0)	75 (17.6)

[a] Participants' numbers vary due to missing data.

© 2019 Coll CVN et al. *JAMA Network Open.*

eTable 2. Mean postpartum EPDS score and the proportion of women with a positive screening for postpartum depression by study group (multiple imputation). [a]

Outcomes	Intention-to-treat			
	Intervention (n=213)	Control (n=426)	Treatment effect (95% CI)	p value
	mean ± SD or N (%)			
EPDS (score)	4.9 ± 3.7	5.4 ± 4.1	- 0.5 (-1.2, 0.2)	0.14
EPDS ≥12	12 (6.2)	36 (9.1)	0.67 (0.34, 1.30)	0.24

[a] Missing values in the EPDS score at the 3 months postpartum follow-up were replaced by imputed values using chained equations with the predicted mean matching method based on the baseline explanatory variables of postpartum EPDS scores (age, marital status, skin color, parity, planned pregnancy, previous depression, pre-pregnancy physical activity, smoking in the first trimester of pregnancy, family income, schooling and pre-pregnancy BMI) and group assignment. We created forty imputed datasets and used the same model as above to estimate the intervention effects on postpartum depression in these datasets.

© 2019 Coll CVN et al. *JAMA Network Open.*
