# Supplementary material for: Efficacy of Regular Exercise During Pregnancy on the Prevention of Postpartum Depression: The PAMELA Randomized Clinical Trial
Source: JAMA Netw Open. 2019 Jan 4;2(1):e186861. doi: 10.1001/jamanetworkopen.2018.6861 (PMC6324311; doi:10.1001/jamanetworkopen.2018.6861)
Supplement: Supplement 3. — Data Sharing Statement [file jamanetwopen-2-e186861-s003.pdf]

Data Sharing Statement

Coll. Efficacy of Regular Exercise During Pregnancy on the Prevention of Postpartum Depression. *JAMA Netw Open*. Published January 04, 2019. 10.1001/jamanetworkopen.2018.6861

Data
Data available: No

Additional Information
Explanation for why data not available: Data available upon request
